# Attention-Based Recurrent Temporal Restricted Boltzmann Machine for Radar High Resolution Range Profile Sequence Recognition

**DOI:** 10.3390/s18051585

**Published:** 2018-05-16

**Authors:** Yifan Zhang, Xunzhang Gao, Xuan Peng, Jiaqi Ye, Xiang Li

**Affiliations:** College of Electronic Science, National University of Defense Technology, Changsha 410073, China; zhangyifan16@nudt.edu.cn (Y.Z.); pengxuan@nudt.edu.cn (X.P.); 18390906478@163.com (J.Y.); lixiang01@vip.sina.com (X.L.)

**Keywords:** HRRP, RATR, RTRBM, attention mechanism

## Abstract

The High Resolution Range Profile (HRRP) recognition has attracted great concern in the field of Radar Automatic Target Recognition (RATR). However, traditional HRRP recognition methods failed to model high dimensional sequential data efficiently and have a poor anti-noise ability. To deal with these problems, a novel stochastic neural network model named Attention-based Recurrent Temporal Restricted Boltzmann Machine (ARTRBM) is proposed in this paper. RTRBM is utilized to extract discriminative features and the attention mechanism is adopted to select major features. RTRBM is efficient to model high dimensional HRRP sequences because it can extract the information of temporal and spatial correlation between adjacent HRRPs. The attention mechanism is used in sequential data recognition tasks including machine translation and relation classification, which makes the model pay more attention to the major features of recognition. Therefore, the combination of RTRBM and the attention mechanism makes our model effective for extracting more internal related features and choose the important parts of the extracted features. Additionally, the model performs well with the noise corrupted HRRP data. Experimental results on the Moving and Stationary Target Acquisition and Recognition (MSTAR) dataset show that our proposed model outperforms other traditional methods, which indicates that ARTRBM extracts, selects, and utilizes the correlation information between adjacent HRRPs effectively and is suitable for high dimensional data or noise corrupted data.

## 1. Introduction

A high-resolution range profile (HRRP) is the amplitude of the coherent summations of the complex time returns from target scatters in each range cell, which represents the projection of the complex returned echoes from the target scattering centers on to the radar line-of-sight (LOS) [1]. The HRRP recognition has been studied for decades in the field of RATR because it contains important structural information such as the target size and the distribution of scattering points [1,2,3,4]. In addition, the HRRP is easy to obtain, store, and process. For the problem of HRRP recognition, a large number of scholars have conducted extensive research [1,5,6,7]. The reported methods can be summarized as extracted features of HRRPs after dividing the full target radar aspect angles into several frames and performing the target detection to select the region of interest in an HRRP. The difference between these methods lies in feature extraction. Common feature extraction techniques include HRRP templates, HRRP stochastic modeling, time-frequency transform features, and invariant features [8,9]. These feature extraction techniques all have clear physical meaning and are conducive for promotion. However, most traditional recognition methods utilize the single HRRP rather than HRRP sequences, which ignores the temporal and spatial correlation within the sample.

Noting strong relativity is contained between the adjacent HRRP, sequential HRRP is of potential usage for recognition. To make use of the spatial and temporal correlation in a sequence, HMM is often utilized for sequential problems such as sequential event detection in wireless sensor networks and radar HRRP sequence recognition [10,11]. This method utilizes the sequence information of HRRP and considers the structure information inside the target. In addition, the problem of azimuth sensitivity is solved by framing [12,13,14]. However, the model can only represent local dependencies between states and has a high computational complexity, which means it is not efficient at dealing with high dimensional sequential data. Recently, deep learning has been gradually applied to radar. Ahmet Elbir constructed a CNN model as a multi-class classification framework to select antennas in a cognitive radar scenario, which is an essential application of deep learning in the radar field [15]. However, the provided method still does not consider the situation of high dimensional sequential data.

Dealing with high dimensional sequential data has also been widely studied in the machine learning community. Recently, a time-series model, which is potentially better studied to capture dependency structures, relies on the use of Recurrent Neural Networks (RNNs). However, there are many parameters that need to be trained in the model, which leads to the problem of gradient dissipation or gradient explosion in the training process [16]. The Residual Network (ResNet) can effectively solve the problem of gradient dissipation or gradient explosion by sharing the cross layer parameter and retaining the intermediate features [17]. However, the model has no obvious advantages in the processing of sequential data. Following the invention of the fast learning algorithm named contrastive divergence algorithm (CD) [18] and its successful application to Restricted Boltzmann Machine (RBM) learning, the Recurrent Temporal Restricted Boltzmann Machine (RTRBM) has been proposed as a generative model for high dimensional sequences [19,20,21,22,23,24]. More specifically, the RTRBM model is constructed by rolling multiple RBMs over time [21] where each RBM has a contextual hidden state that is received from the previous RBM and is used to modulate its hidden units. Add to it, RBM is a bipartite graphical model that uses a layer of “hidden” binary variables or units to model the probability distribution of a layer of “visible” variables [24,25,26,27,28]. Based on this, the RTRBM model introduces the correlation matrix between the hidden layers of adjacent RBMs to tack the correlation inside the data into consideration [19]. The model has achieved great success in extracting internal correlations between adjacent HRRPs and capturing spatial and temporal patterns in highly dimensional sequential data. In the traditional method based on RTRBM, only one hidden layer (at time frame t) is utilized for the recognition. However, in the training process of the RTRBM model, the gradient of the parameters is propagating with time series, the ‘vanishing gradient problem’ appears easily when T becomes longer. Therefore, with the time series propagating, the model cannot extract deeper features and the sequential correlation features cannot transmit to the next RBM smoothly in the learning process. As such, it is necessary to consider feature vectors at all the T time sequences. Considering that the contribution of each feature vector to the recognition is different and has been ignored in the traditional method based on RTRBM, it is essential for the recognition method to gain the ability to pay more attention to the important feature parts. 

In order to solve the problems which have been put forward, a new method that combines the RTRBM model with the attention mechanism [29] for sequential radar HRRP recognition is proposed in this paper. The attention mechanism was first proposed in the field of the visual image in Reference [30] and has shown good performance on a range of tasks including machine translation, machine comprehension, and Relation classification [31,32,33,34,35,36]. Therefore, it is theoretically possible for HRRP sequence recognition when utilizing the attention mechanism. In ARTRBM, the combination of RTRBM and the attention mechanism makes the model focus its attention on specific features, which are important for the classification task. More specifically, this model encodes the HRRPs sequence through the RTRBM model and then calculates the weight coefficient for each hidden unit, according to their contributions to the recognition performance. Then the features are utilized to construct the attention layer for the recognition task. This combination brings performance improvements for high recognition accuracy achievement and strong robustness to noise. To verify the effectiveness of the proposed model, two experiments are executed, which utilizes the HRRP data converted from the SAR data of MSTAR [37]. Experimental results indicate the superior performance of the proposed model against HMM, Class RBM, and Principle Component Analysis (PCA). Additionally, the proposed model can still achieve an ideal accuracy when the intensity of noise is lower than −15, which confirms its strong robustness to noise.

This paper is organized as follows. In Section 2, the RBM and RTRBM are briefly introduced as a preparation for the proposal of the method. In Section 3, the proposed model for sequential HRRP recognition is presented in detail, which is followed by the training method for the proposed model in Section 4. After that, several experiments on the MSTAR dataset have been performed to evaluate our model in Section 5. Lastly, we conclude our work in Section 6.

## 2. Preliminaries

In this section, we will go over the salient properties of the Restricted Boltzmann Machine (RBM) briefly and then give preliminaries about Recurrent Temporal Restricted Boltzmann Machine (RTRBM), which is a temporal extension of RBMs.

### 2.1. Restricted Boltzmann Machine

**Figure 1:**
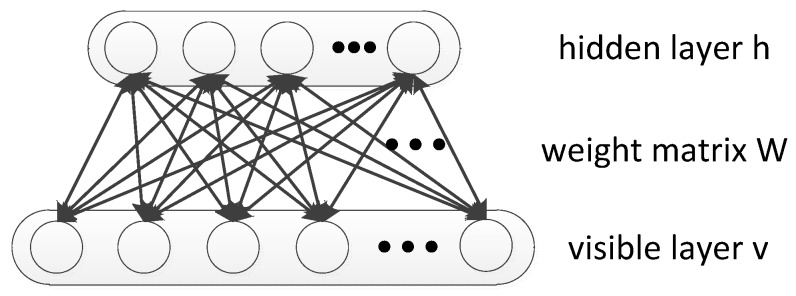
Graphical depiction of the RBM.

The RBM is an undirected graphical model that uses a layer of hidden variables $h=\left[h_{1},h_{2},\cdots h_{m}\right]$ to model a joint distribution over the visible variables $v=\left[v_{1},v_{2},\cdots v_{n}\right]$ [16]. The graphical depiction of the RBM is depicted in Figure 1. The two layers are fully connected to each other by a weight matrix W but there exists no connections between units within the same layer [28,38]. On the problem of HRRP-based RATR, visible units can be an HRRP sample and the hidden layer can be used to extract the features.

The RBM defines the joint distribution over visible units v and hidden units h, which is shown in the equation below [24].

(1) $$p\left(v,h\right)=\frac{\exp\left[-E\left(v,h\right)\right]}{Z}$$

where $Z=\underset{v}{\sum }\underset{h}{\sum }\exp\left[-E\left(v,h\right)\right]$ is the partition function, which is given by adding all possible pairs of visible and hidden vectors. Additionally, E is an energy function defined below.

(2) $$E\left(v,h\right)=-h^{T}\mathrm{Wv}-b^{T}v-c^{T}h$$

where Θ={W,b,c} consists of the model parameters, $W\in R^{M\times N}$ represent the weight matrix connecting visible and hidden vectors, and $b\in R^{N}$ and $c\in R^{M}$ are the biases of the visible and hidden layers, respectively.

### 2.2. Recurrent Temporal Restricted Boltzmann Machine

The Recurrent Temporal Restricted Boltzmann Machine is a generative model for modeling high-dimensional sequences, which was constructed by rolling multiple RBMs over time. In detail, the RBM at time step t is connected at t − 1 through the weight matrix $W_{\mathrm{hh}}$ and is conditioned on it. The dependency on ${\hat{h}}^{\left(t\right)}$ is the major difference compared to the RBM. It is worth noting that this horizontal deep architecture is different from the Deep Brief Networks (DBN), which stacks RBMs vertically [39]. Therefore, more sequence information can be extracted by RTRBM and performs better in many application scenarios such as radar HRRP target recognition.

**Figure 2:**
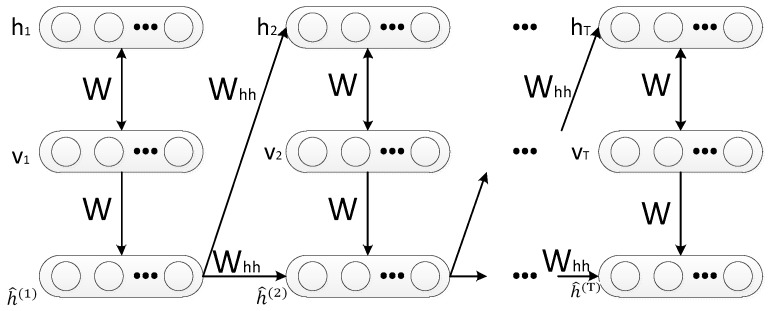
Graphical structure of the RTRBM.

The graphical model for the RTRBM is illustrated in Figure 2. 

The model gives five parameters $\left\{W,W_{\mathrm{hh}},{\hat{h}}^{\left(t\right)},b,c\right\}$. Here W is the weight matrix between the visible and the hidden layer of the RBM at each time frame. $W_{\mathrm{hh}}$ stands for the directed weights, which connect the hidden layer at time t – 1 and t, and ${\hat{h}}^{\left(t\right)}$ is a vector of initial mean-file values of the hidden units. The motivation for the choice of ${\hat{h}}^{\left(t\right)}$ is that, using the RBM associated with time instant t, we have that $E\left(h^{\left(t\right)}|v^{\left(t\right)}\right)={\hat{h}}^{\left(t\right)}$; i.e., it is the expected value of the hidden units vector. In addition, $b^{\left(t\right)}$ and $c^{\left(t\right)}$ are the biases of visible and hidden layers. respectively. In RTRBM, the RBM at time frame t is conditioned on itself at time step t − 1 through a set of time dependent model parameters such as the visible and hidden layer biases $b^{\left(t\right)}$ and $c^{\left(t\right)}$ that depend on ${\hat{h}}^{\left(t-1\right)}$ [40].

(3) $$\{\begin{array}{c} b^{\left(t\right)}=W_{\mathrm{hh}}{\hat{h}}^{\left(t-1\right)}+b\\ c^{\left(t\right)}=W_{\mathrm{hh}}{\hat{h}}^{\left(t-1\right)}+c \end{array}$$

while ${\hat{h}}^{\left(t\right)}$ is the mean-filed value of $h^{\left(t\right)}$, which is represented in detail below.

(4) $${\hat{h}}^{\left(t\right)}=\sigma \left({\mathrm{Wv}}^{\left(t\right)}+c^{\left(t\right)}\right)=\{\begin{array}{ll} \sigma \left({\mathrm{Wv}}^{\left(t\right)}+c_{0}\right) & \mathrm{if}\;t=1;\\ \sigma \left({\mathrm{Wv}}^{\left(t\right)}+W_{\mathrm{hh}}{\hat{h}}^{\left(t-1\right)}+c\right) & \mathrm{if}\;t>1. \end{array}$$

Given hidden inputs ${\hat{h}}^{\left(t-1\right)}$(t > 1), the conditional distributions are factorized and takes the form below.

(5) $$\{\begin{array}{l} P\left(h_{t,j}=1|v,{\hat{h}}^{\left(t-1\right)}\right)=\sigma \left(\underset{i}{\sum }\omega_{j,i}v_{t,i}+b_{j}+\underset{l}{\sum }W_{{\mathrm{hh}}_{j,m}}{\hat{h}}^{\left(t-1,m\right)}\right)\\ P\left(v_{t,i}=1|h_{t},{\hat{h}}^{\left(t-1\right)}\right)=\sigma \left(\underset{i}{\sum }\omega_{j,i}h_{t,j}+c_{i}\right) \end{array}$$

Therefore, the joint probability distribution of the visible and hidden units of the RTRBM with length T takes the form below [21].

(6) $$p\left(v^{\left(1:T\right)},h^{\left(1:T\right)};{\hat{h}}^{\left(1:T-1\right)}\right)=\overset{T}{\underset{t=1}{\prod }}p\left(v^{\left(t\right)},h^{\left(t\right)};{\hat{h}}^{\left(t-1\right)}\right)=\overset{T}{\underset{t=1}{\prod }}\frac{\exp\left[-E\left(v^{\left(t\right)},h^{\left(t\right)};{\hat{h}}^{\left(t-1\right)}\right)\right]}{Z_{{\hat{h}}^{\left(t-1\right)}}}$$

where $Z_{{\hat{h}}^{\left(t-1\right)}}$ denotes the normalization factors for the RBM at T = t and $E\left(v^{\left(t\right)},h^{\left(t\right)},{\hat{h}}^{\left(t-1\right)}\right)$ is the energy function at the time step t, which is defined by the equation below.

(7) $$E\left(v^{\left(t\right)},h^{\left(t\right)};{\hat{h}}^{\left(t-1\right)}\right)=-h^{\left(t\right)T}\mathrm{Wv}-c^{\left(t\right)T}v-b^{\left(t\right)T}h^{\left(t\right)}$$

Furthermore, given the hidden inputs ${\hat{h}}^{\left(1\right)},{\hat{h}}^{\left(2\right)},\cdots ,{\hat{h}}^{\left(T\right)}$, all the RBMs are decoupled. Therefore, sampling can be performed using block Gibbs sampling for each RBM independently. This fact is useful in deriving the CD algorithm, which is a stochastic approximation and utilizes a few Gibbs sampling steps to estimate the gradient of parameters [18,41].

## 3. The Proposed Model

**Figure 3:**
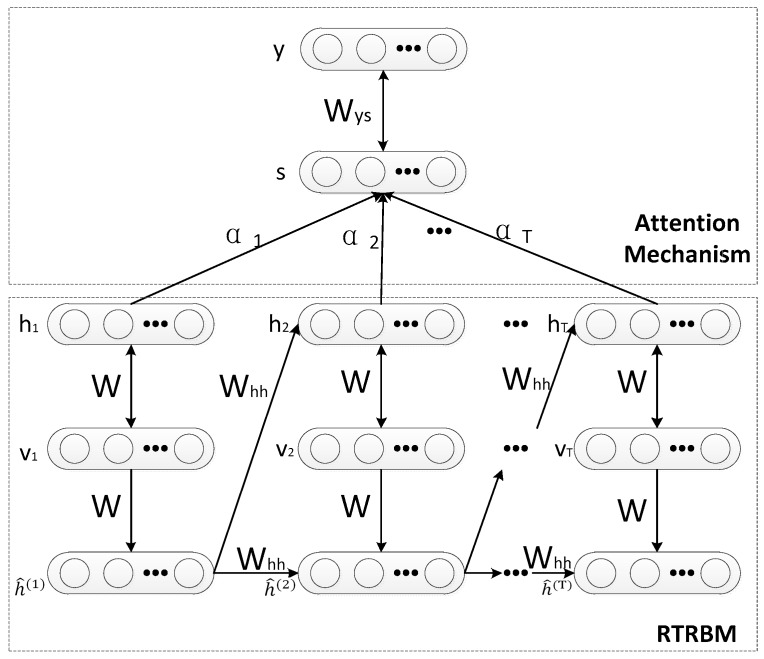
Graphical structure of Attention-based RTRBM.

Based on the original RTRBM, the newly proposed model brings the idea of the attention mechanism, which is named Attention based RTRBM. The graphical structure of the proposed model is demonstrated in Figure 3. In the proposed model, RTRBM is utilized to extract features from the input data and store the extracted features in the hidden vector. A new hidden layer s is introduced to RTRBM by the weighted sum in all hidden layers for the reason of measuring the role of each hidden vector in recognition tasks and then the new hidden layer is used for classification.

In the context of radar HRRP recognition, the input data $v=\left[v_{1},v_{2},\cdots ,v_{N}\right]$ is the raw HRRPs sequence and the output y is a sequence of the class label. Each feature vector is extracted from the RTRBM, which is treated as an encoder to form a sequential representation.

The upper half of Figure 3 represents the attention mechanism in the ARTRBM model. The fundamental principle of the attention mechanism can be expressed as the classifier paying more attention to the major part rather than all the extracted feature vectors. 

As is shown in Figure 3, $\alpha_{t}$ stands for the weight coefficient for the hidden layer at time step t. The layer s is determined by the hidden layer of each time step and $W_{\mathrm{ys}}$ corresponds to the weight matrix, which connects the layer s and output layer y. Additionally, y is a vector representing the class label in which all values are set to 0 except at the position corresponding to a label y, which is set to 1.

In order to detail and describe the process of our model, the flowchart about ARTRBM is shown below.

**Figure 4:**
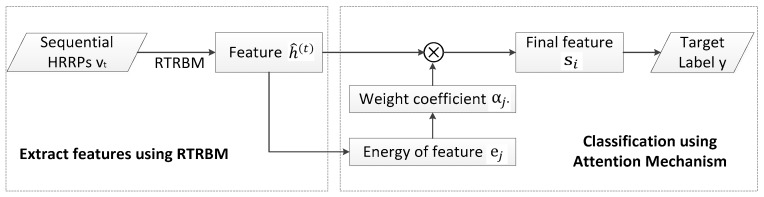
The process of Attention-based RTRBM.

As shown in Figure 4, the basic process of the attention mechanism can be summarized in three steps. First, computing the feature energy $e_{j}$ and weight coefficients $\alpha_{j,}\;$which represent the contribution of extracted feature vectors for recognition. Afterward, the final hidden layer s is constructed, which is determined by the hidden layers of all time steps. Finally, the layer s is used in the final classification task. 

In the attention mechanism, the final feature vector s is obtained by the weighted summation of the hidden layers of each time, which can be expressed in the equation below.

(8) $$s_{i}=\overset{T}{\underset{j=1}{\sum }}\alpha_{j\cdot }\cdot h_{\mathrm{ij}}$$

where the weight coefficient $\alpha_{j\cdot }$ can be defined as:

(9) $$\alpha_{j\cdot }=\frac{\exp\left(e_{j}\right)}{\sum_{j=1}^{T}\exp\left(e_{j}\right)}$$

where $\alpha_{j\cdot }$ represents the vector of the jth row elements of the matrix α and $e_{j}=\mathrm{Va}\cdot \tan h\left(\mathrm{Wa}\cdot h_{j}\right)$ corresponds to the hidden layer energy at time frame j. The weight coefficient $\alpha_{j}$ represents the role of the hidden layer feature $h_{j}$ in recognition. The attention mechanism [30,41,42] is also determined by the parameter $\alpha_{j}$. By training the parameters Va and Wa, the model can assign the hidden layer $h_{j}$ with different weights at different moments, which makes the model more focused on the parts that play a major role in the recognition tasks.

## 4. Learning the Parameters of the Model

In the proposed model, the RTRBM plays a role of the encoder, which describes the joint probability distribution $p\left(v^{\left(1:T\right)},h^{\left(1:T\right)};{\hat{h}}^{\left(t-1\right)}\right)$. According to Equation (3) and Equation (7), the energy function can be computed and is shown below.

(10) $$E\left(v^{\left(1:T\right)},h^{\left(1:T\right)};{\hat{h}}^{\left(1:T-1\right)}\right)=-\left(h_{1}^{T}{\mathrm{Wv}}_{1}+c^{T}v_{1}+b_{0}^{T}h_{1}\right)-\overset{T}{\underset{t=2}{\sum }}\left(h_{t}^{T}{\mathrm{Wv}}_{t}+c^{T}v_{t}+b^{T}h_{t}+h_{t}^{T}W_{\mathrm{hh}}{\hat{h}}_{t-1}\right)$$

In order to learn the parameters, first, we need to obtain the partial derivatives of $\log P(v_{1},v_{2},\cdots ,v_{T})$ with respect to the parameters. We use CD approximation [15,17] to compute these derivatives, which require the gradients of energy function (10) to be based on all the model parameters. Afterward, we separate the energy function into the following two terms $E=-H-Q_{2}$, where:

(11) $$\left\{ \begin{array}{l} H=\left(h_{1}^{T}{\mathrm{Wv}}_{1}+c^{T}v_{1}+b_{0}^{T}h_{1}\right)+\overset{T}{\underset{t=2}{\sum }}\left(h_{t}^{T}{\mathrm{Wv}}_{t}+c^{T}v_{t}+b^{T}h_{t}\right)\\ Q_{2}=\overset{T}{\underset{t=2}{\sum }}\left(h_{t}^{T}W_{\mathrm{hh}}{\hat{h}}_{t-1}\right) \end{array}\right.$$

Therefore, the gradients of E representing the parameters were separated into two parts. It is straightforward to calculate the gradients of $\frac{\partial H}{\partial \Theta }$, and calculating $\frac{\partial Q_{2}}{\partial \Theta }$ would be more complex. To compute $\frac{\partial Q_{2}}{\partial \Theta }$, we first compute $\frac{\partial Q_{2}}{\partial {\hat{h}}^{\left(t\right)}}$, which can be computed recursively using the back propagation-through-time (BPTT) algorithm (David Rumelhart, Geoffrey Hinton et al., 1986) and the chain rule. Therefore, the model parameters Θ can be updated via gradient ascent, which is shown in the equation below.

(12) $$\frac{\partial E}{\partial \Theta }=\frac{\partial \left(H+Q_{2}\right)}{\partial \Theta }=E_{{\left\{h_{t}\right\}}_{t=1}^{T}|{\{v_{t},{\hat{h}}_{t}\}}_{t=1}^{T}}\left[\frac{\partial H}{\partial \Theta }\right]-E_{{\left\{h_{t,}v_{t}\right\}}_{t=1}^{T}|{\left\{{\hat{h}}_{t}\right\}}_{t=1}^{T}}\left[\frac{\partial H}{\partial \Theta }\right]+\frac{\partial Q_{2}}{\partial \Theta }$$

where $E_{{\left\{h_{t}\right\}}_{t=1}^{T}|{\left\{v_{t},{\hat{h}}_{t}\right\}}_{t=1}^{T}}\left[\frac{\partial H}{\partial \Theta }\right]$ represents the universal mean of the gradient function $\frac{\partial H}{\partial \Theta }$ under the conditional probability $p\left({\left\{h_{t}\right\}}_{t=1}^{T}|{\left\{v_{t},{\hat{h}}_{t}\right\}}_{t=1}^{T}\right)$ and can be expressed using the equation below.

(13) $$E_{{\left\{h_{t}\right\}}_{t=1}^{T}|{\left\{v_{t},{\hat{h}}_{t}\right\}}_{t=1}^{T}}\left[\frac{\partial H}{\partial \Theta }\right]=\sum_{t=1}^{T}p\left(h_{t}|v_{t},{\hat{h}}_{t}\right)\cdot \frac{\partial H}{\partial \Theta }$$

Therefore, Equation (12) can be derived as:

(14) $$\frac{\partial E}{\partial \Theta }=\frac{\partial \left(H+Q_{2}\right)}{\partial \Theta }=\sum_{t=1}^{T}p\left(h_{t}|v_{t},{\hat{h}}_{t}\right)\cdot \frac{\partial H}{\partial \Theta }-\sum_{t=1}^{T}p\left(h_{t,}v_{t}\right)\cdot \frac{\partial H}{\partial \Theta }+\frac{\partial Q_{2}}{\partial \Theta }$$

Specifically, $\frac{\partial H}{\partial \Theta }$ and $\frac{\partial Q_{2}}{\partial \Theta }$ are shown in Appendix A.

We extract the features from the input data with the RTRBM model, which are stored in $h^{\left(j\right)}$ at every time step. Then we use $h^{\left(j\right)}$ as the input for the attention mechanism and compute the final hidden layer s using Equation (8). To learn the parameters of the attention mechanism, we need to choose an appropriate objective function. Here we use a close variant of perplexity known as cross entropy, which represents the divergence between the entropy calculated from the predicted distribution and that of the correct prediction label (and can be interpreted as the distance between these two distributions). It can be computed using all the units of the layer s and expressed as:

(15) $$f_{\mathrm{Cross}}\left(\theta ,D_{\mathrm{train}}\right)=-\frac{1}{\left|D_{\mathrm{train}}\right|}\overset{\left|D_{\mathrm{train}}\right|}{\underset{n=1}{\sum }}\ln p\left(y^{n}|s^{n}\right)$$

where $D_{\mathrm{train}}=\left\{\left(s^{n},y^{n}\right)\right\}$ is the set of training examples, n represents the serial number of the training sample, and $s^{n}=\left(s_{1}^{n},s_{2}^{n},\cdots s_{T}^{n}\right)$ is the final hidden layer while $y^{n}=\left(y_{1}^{n},y_{2}^{n},\cdots y_{T}^{n}\right)$ corresponds to the target labels. By taking Equations (8) and (9) into the objective function (15), the gradient $\frac{\partial }{\partial \theta }f_{\mathrm{Cross}}\left(\theta ,D_{\mathrm{train}}\right)$ can be calculated and is derived below.

(16) $$\frac{\partial }{\partial \theta }f_{\mathrm{Cross}}\left(\theta ,D_{\mathrm{train}}\right)=\frac{1}{\left|D_{\mathrm{train}}\right|}\overset{\left|D_{\mathrm{train}}\right|}{\underset{n=1}{\sum }}\frac{\partial F\left(y^{n}|s^{n}\right)}{\partial \theta }$$

where:

(17) $$F\left(y^{n}|s^{n}\right)=-\ln\underset{c_{i}}{\sum }p\left(y^{n}|s^{n}\right)$$

and:

(18) $$p\left(y^{n}|s^{n}\right)={y\mathrm{lny}}^{\prime }-\left(1-y\right)\ln\left(1-y^{\prime }\right)$$

with:

(19) $$y^{\prime }=\sigma \left(W_{\mathrm{ys}}\cdot s+d\right)$$

where y and y′ denotes the correct label and the output label, respectively. $W_{\mathrm{ys}}$ is the weight matrix that connects layer s and label vector y while the logic function $\sigma \left(x\right)={\left(1+\exp\left(-x\right)\right)}^{-1}$ is applied to each element of the argued vector. Therefore, the gradients $\frac{\partial F\left(y^{n}|s^{n}\right)}{\partial \theta }$ can be exactly computed. The brief deduction process and results are show in Appendix B.

The pseudo code of the model parameter update for the proposed model is summarized in Algorithm 1, which is shown below.

**Algorithm 1.** Pseudo code for the learning steps of Attention based RTRBM model**Input:** training pair: {v_train; y_train}, hidden layer size: dim_h; learning rate: $\lambda_{1},\lambda_{2}$; momentum: β; and weightcost: $\eta_{1},\eta_{2}$.**Output:** label vector y# **Section 1: Extract features using RTRBM**
 (1): Calculate ${\hat{h}}^{\left(t\right)}$ according to Equation (4). (2): Calculate $P\left(h_{t,j}=1|v,{\hat{h}}^{\left(t-1\right)}\right)$ and $P\left(v_{t,i}=1|h_{t},{\hat{h}}^{\left(t-1\right)}\right),$ respectively,according to Equation (5). (3): Calculate the L2 reconstruction error: $\mathrm{Loss}\leftarrow {∥v}_{t}-v_{t}\_{k∥}_{2}$. (4): Update parameters of this section: Θ←Θ−ΔΘ, $\Delta \Theta \leftarrow \beta \Delta \Theta -\lambda_{1}\left(\nabla \Theta -\eta_{1}\Theta \right)$ (5): Repeat step (1) to (4) for 1000 epochs and save the trained Θ for test phase. # **Section 2: Classification with Attention mechanism**(1): Calculate $\alpha_{j},j\in \left(1,2,\cdots ,T\right)$ according to Equation (9).(2): Calculate $s_{i},i\in \left(1,2,\cdots ,\dim\_h\right)$ according to Equation (8).(3): Calculate the cross entropy according to Equation (15).(4): Update parameters of this section: $\theta \leftarrow \theta -\lambda_{2}\left(\nabla \theta -\eta_{2}\theta \right)$(5): Repeat step (1) to (4) for 1000 epochs and save the trained θ for the test phase.

## 5. Experiments

In order to evaluate the proposed recognition model, several experiments on the MSTAR dataset have been presented. First, arranging the training and testing HRRP sequences was introduced in Section 5.1. Afterward, we completed two experiments with different purposes in Section 5.2. The first section compared the performance of our proposed model with several other comparative models and the second section tested the recognition ability of our model with different noise intensities.

### 5.1. The Dataset

**Figure 5:**
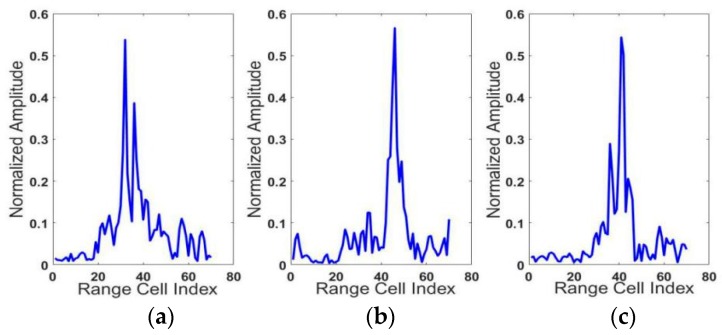
HRRPs of the three targets. (**a**) BMP2(Sn_C9563), (**b**) T72(Sn-132), (**c**) BTR70(Sn_71).

In order to show the clear comparisons between our results with those in other papers more easily, the publicly-available MSTAR (Moving and Stationary Target Acquisition and Recognition) dataset, which has been widely used in related research was chosen in our experiments [12]. MSTAR is funded by DARPA and is the standard dataset of the SAR automatic target recognition algorithm. More detailed, the MASTAR dataset includes 10 kinds of targets data (X band) under different azimuth angles and we chose three of the most similar targets for the experiment, which are the T72 main battle tank, the BMP2 armored personal carrier, and the BTR70 armored personal carrier. In order to make the MSTAR dataset suitable for our model, we first transformed the two-dimensional SAR into a one-dimensional HRRP vector to train our proposed model. The HRRP of the three targets are shown in Figure 5.

All three classes of targets cover 0 to 360 degrees of aspect angles and their distance and azimuth resolutions are 0.3 m [43,44]. In the dataset, each target is obtained under the depression angle of 15° and 17°. The HRRPs of 17 degree of depression angle were used as the training data while the HRRPs of 15° were used as the test data. The size of the training and testing dataset is briefly illustrated in Table 1. 

We can see from the table that there are three targets in the table. The targets BMP2 and T72 contain three similar models, respectively, while BTR70 contains one model. Taking BMP2 as an example, we use Sn_C9563 to train the ARTRBM model and test it with Sn_C9563, Sn_C9566, and Sn_C21. In this way, the generalization performance of our model can be examined. The training set and testing set contain 6980 HRRPs and 13,650 HRRPs, respectively. 

**Figure 6:**
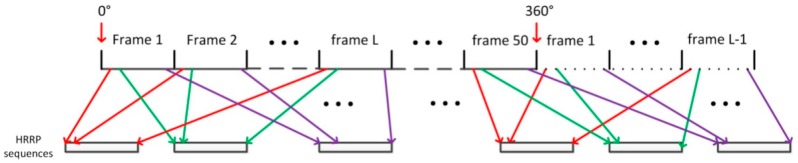
The composition of the sequential HRRP datasets.

We divided the 360° of aspect angles into 50 aspect frames uniformly. Each frame covers 7.2°. In each frame, an HRRP is sampled at intervals of 0.1 degrees. Therefore, each frame contains 72 HRRPs. Additionally, the composition of the sequential HRRP datasets is shown in Figure 6.

To make the process more clearly, suppose that each HRRP sequence contains L(L≥T) HRRPs and the steps to construct the sequential HRRP are shown as Algorithm 2 [45]. 

**Algorithm 2.** The composition of the sequential HRRP datasets.Step 1: Start from the aspect frame 1 to L. The first HRRPs in frame 1 to L are chosen to form the first HRRP sequence with length L. Slide one HRRP to the right and the second HRRPs in aspect frame 1 to L are chosen to form the second HRRP sequence. Repeat this algorithm until the end of each frame.Step 2: Slide one frame to the right and repeat step 1 to construct the following sequences.Step 3: Repeat step 2 until the end of all aspect frames. If the remaining frame is less than L, then the first L−1 frames are cyclically used one by one to form the remaining sequences.

In many studies, the clutter is removed to get “clean” HRRPs. We directly used the raw HRRPs. The only preprocessing was normalizing the magnitude of each HRRP to its total energy. This setting could make the experiments more closed to real recognition scenarios.

### 5.2. Experiments

#### 5.2.1. Experiment 1: Investigating the Influence of Hidden Layer Size on Recognition Performance

In this experiment, we will investigate the influence of the size of the hidden layer on recognition performance. In order to explore this problem, two groups of contrastive experiments were organized for different purposes. The first group is aimed at comparing the performance of the Attention-based RTRBM model with contrast models on different hidden layer sizes while the second is to investigate whether the attention mechanism really works and how much effect it has on performance.

Before conducting the experiments, we analyzed the influence rising from the length of the RTRBM model at first. According to Table 2, it shows that when T is increased by more than 15, stable test accuracy can be achieved. In addition, we can further improve the recognition rate by adding hidden units. Therefore, to seek a balance between recognition accuracy and computational complexity, T = 15 is adopted for the recognition task.

(A) Comparing the Performance of the Proposed Model with the Traditional Models

**Figure 7:**
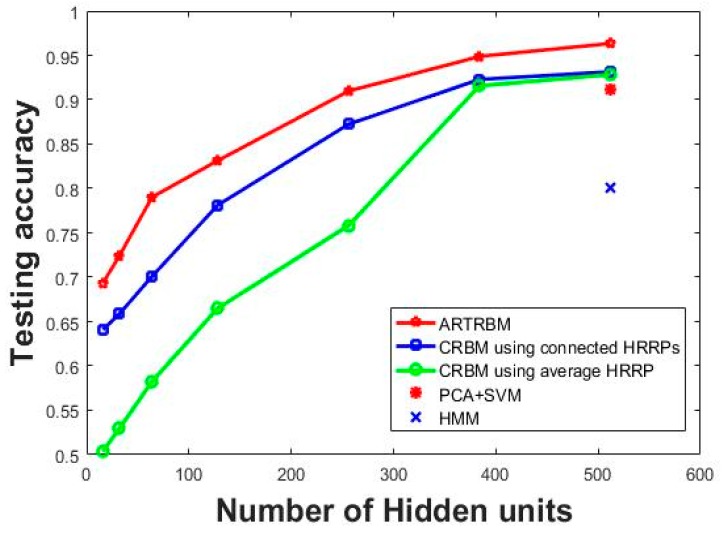
The recognition performance on five models with a different number of hidden units at T = 15.

In the first group of contrast experiment, Class RBM (CRBM) with different hidden layer sizes (number of hidden nodes = 16, 32, 64, 128, 256, 384, 512) were trained as comparisons to the proposed method. We carry out the contrast experiments with two different data input methods by constructing an average HRRP with 15 adjacent HRRPs and connecting 15 HRRPs end-to-end. The recognition performance of each model is shown in Figure 7 where the test accuracy is computed by averaging the test results of the three targets.

It can be seen in Figure 7 that the superior recognition performance of Attention-based RTRBM against the other two models. Additionally, our proposed model gets optimal recognition accuracy on each size of the hidden layer, which shows the strong ability to deal with high dimensional sequences. The explanation for this result is that the proposed model can extract more separable features through the RTRBM model and make better use of them using the attention mechanism. Class RBM with average HRRP performs not as good as the other two models, but gets ideal recognition accuracy when the number of hidden nodes increased to 384, which reflects that Class RBM needs more hidden units to reach high recognition accuracy. 

We design another baseline using PCA to reduce the dimension of input data. There are 15 features retained after PCA and the classifier is the Support Vector Machine (SVM). We repeat the baseline five times and the average test accuracy is 91.22%. Since the contrast experiment PCA+SVM does not contain hidden units, we mark the results of the model at 512 hidden units in Figure 7. Therefore, we can compare the PCA+SVM model with the best results of other methods. Additionally, the test performance of the HMM model is lower than 80% when the sequence length is 15, which is provided by Reference [12]. Similarly, we mark the results HMM at 512 hidden units in Figure 7 to compare with the best results of other methods. Then we can conclude from Figure 7 that the correlation matrix between the adjacent hidden layers helps RTRBM to extract more discriminatory features and the weight coefficients make the attention mechanism select more separable features, which means that ARTRBM is more suitable for the radar HRRP sequence recognition task.

To gain insight into the performance of three methods on different targets, we list the confusion matrix for the three targets in Table 3. The number of hidden units for all the methods is 384.

**Figure 8:**
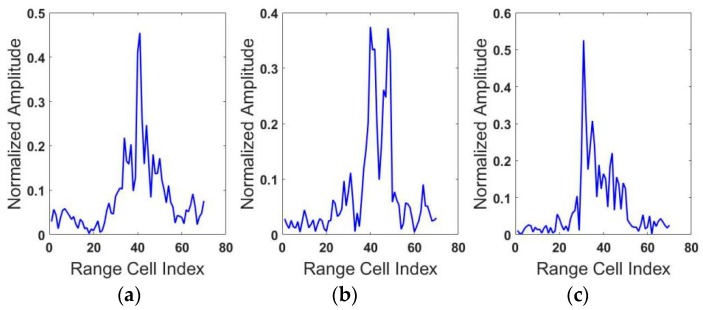
Range profiles of three types of BMP2. (**a**) Sn_C9563, (**b**) Sn_C9566, (**c**) Sn_C21.

As shown in Table 3, the misclassification of BMP2 lowers the average accuracy. One possible reason is that the features learned by the three models are not discriminatory enough to recognize the true targets and another reason may be summarized as we train the models only on BMP2 (Sn_C9563). However, test models on three types of the targets BMP2 and the three types of BMP2 (shown in Figure 8) has a low similarity, which is lower than the three types of T72. However, our proposed model still achieves higher accuracy than two contrast models on the classification of BMP2, which indicates that Attention-based RTRBM is a better choice when there is a great difference between the training and testing dataset. 

(B) Evaluating the Impact of Attention Mechanism on Recognition Performance

In the second contrastive experiments group, we designed several ways in which, without attention mechanism, we complete the comparison. In addition, the purpose is to investigate the impact of attention, which is the mechanism in the recognition performance. 

The feature information are extracted by RTRBM and contained in the hidden layer, which are expressed as ${\hat{h}}^{\left(1\right)},{\hat{h}}^{\left(2\right)},\cdots ,{\hat{h}}^{\left(T\right)}$. We use ${\hat{h}}^{\left(1\right)},{\hat{h}}^{\left(middle\right)},{\hat{h}}^{\left(T\right)},{\hat{h}}^{\left(mean\right)}$ (the feature of the first, middle, last, and the average of all time frames) as input data, respectively, and classify it with a Single Layer Perceptron (SLP) model. In other words, we can regard the baselines as special forms of ARTRBM that set the coefficients to [1,0,⋯,0],[0,⋯,0,1,0,⋯,0],[0,0,⋯,1] and $\left[\frac{1}{T},\frac{1}{T},\cdots ,\frac{1}{T}\right],$ respectively. For fair comparison, in this experiment, T is set to 15 and the number of hidden units is 384, which can achieve an ideal accuracy with low computation complexity. Therefore, ${\hat{h}}^{\left(middle\right)}$ represents the hidden features when t = 8. 

**Figure 9:**
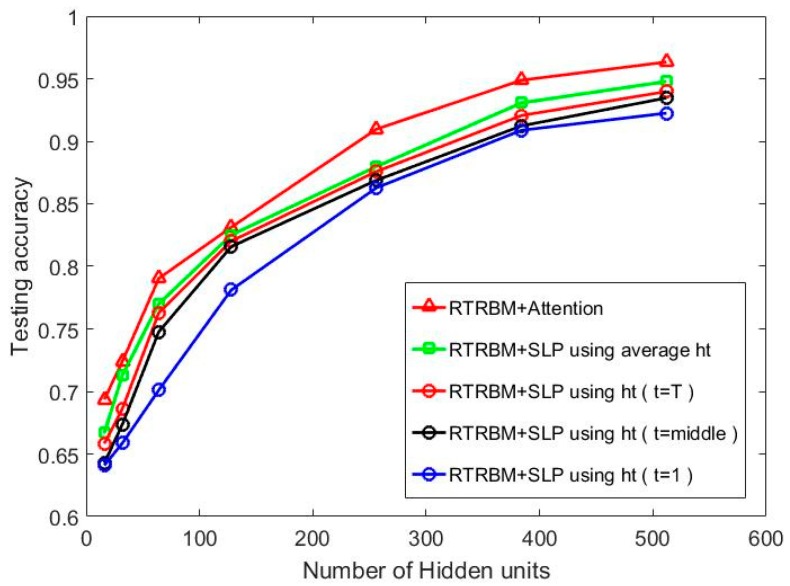
Recognition performance on models trained with features extracted by RTRBM.

As shown in Figure 9, the proposed model achieves higher recognition accuracy than the other four methods at all hidden layer sizes. This result indicates that the attention mechanism can select discriminatory features more efficiently than other methods that select average ${\hat{h}}^{\left(t\right)}$ or any single ${\hat{h}}^{\left(t\right)}$. It is worth noting in the figure that choosing average ${\hat{h}}^{\left(t\right)}$ performs better than the other three contrastive experiments. In addition, with the time step t increases, RTRBM+SLP models perform better. This is not surprising since the latter ${\hat{h}}^{\left(t\right)}$ contains more temporal and spatial correlation information through the correlation matrix $W_{\mathrm{hh}}$. However, even the RTRBM+SLP model using ${\hat{h}}^{\left(T\right)}$ still performs worse as our proposed model. Therefore, the attention mechanism greatly contributes to the recognition performance.

#### 5.2.2. Experiment 2: Investigating the Influence of SNR on Recognition Performance

**Figure 10:**
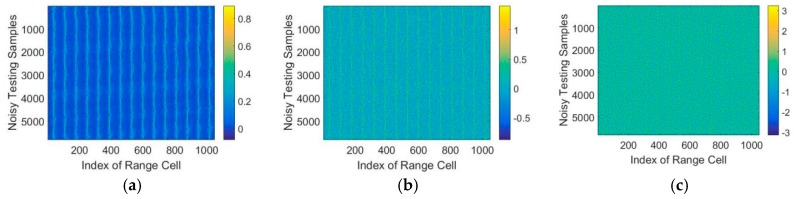
Testing HRRP data with different SNR (**a**) SNR = 20 dB, (**b**) SNR = 10 dB, (**c**) SNR = 5 dB.

For applications in real scenarios, white Gaussian noise of different Signal-to-Noise (SNR) increasing from −10dB to 30dB were added to the testing data to investigate the robustness of the proposed model. In addition, the test data with different SNR are shown in Figure 10.

As shown in Figure 10, white Gaussian noise of different SNRs is superimposed on the test HRRP sequence. Each row in the figure represents the index of range cell while each column shows the number of testing data. We use T72 as example, which contains 5820 HRRP samples.

In this example, we trained the ARTRBM using the HRRP sequence with T = 15 and 384 hidden units. We choose the Class RBM with 384 hidden units as the contrast experiment and the data input method connected 15 HRRPs end to end, which performs better than all other contrastive experiments in Experiment 1. Another contrast experiment uses PCA to reduce the dimension to 15 of input data and the classifier is the Support Vector Machine (SVM).

**Figure 11:**
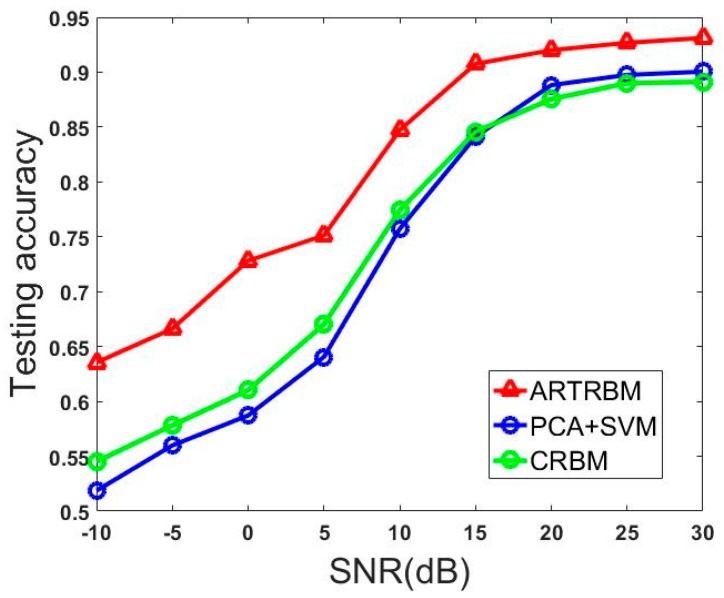
Recognition performance on models tested with different SNR.

Figure 11 shows the recognition performance of three models with different SNR. It is obvious that our proposed model achieves better performance than the other two models at all SNR levels and it gets more than 10% advantage over the other two models at −10dB. Additionally, the testing accuracy keeps stable at a high level, which is near the average accuracy in Table 2 (0.9488) when the SNR is higher than 15dB, which inflects that our proposed model has a certain anti-noise ability. The accuracy of the proposed model decreases to about 65% with the decrease of SNR. However, this number is less than 55% for CRBM. This result shows the strong anti-noise power of ARTRBM. Considering the working environment of the radar system, the training samples are often corrupted by noise. The model we proposed is a better choice to perform the HRRP sequence recognition task.

## 6. Conclusions

In this paper, attention-based RTRBM is proposed for target recognition based on the HRRP sequence. Compared with the reported methods, the proposed method has some compelling advantages. First, it introduces the correlation matrix between the hidden layers to extract more correlation information, which makes the extracted features hold the previous and current information. Afterward, it efficiently deals with high dimensional sequential data, which performs better than Class RBM using two different data input methods. Additionally, it can be effective for choosing and utilizing the important parts of the extracted features, which outperforms the RTRBM+SLP model using different input features. Additionally, the proposed model performs well in the case of strong noise, which indicates a strong robustness for the noise. In the near future, to better solve the problem of sequential HRRP recognition, we plan to combine other deeper models with an attention mechanism as a classifier for RTRBM or other sequential feature extraction models. Furthermore, in order to make the model more applicable to the real scenario, we will operate related experiments in the cases of different waveforms and pulse recurrence intervals (PRIs) or the case of the training phase and testing phase at different angular sampling rates. Additionally, we attempt to develop a model that can set the length of the attention mechanism adaptively. In this case, the number of T will not need to be set by experience, which may achieve a better performance.

## Figures and Tables

**Table 1:** Training and testing set of HRRPs for three targets.

Number	Training Set	Size	Testing Set	Size
1	BMP2 (Sn_C9563)	2330	BMP2 (Sn_C9563)	1950
			BMP2 (Sn_C9566)	1960
			BMP2 (Sn_C21)	1960
2	T72 (Sn_132)	2320	T72 (Sn_132)	1960
			T72 (Sn_812)	1950
			T72 (Sn_S7)	1910
3	BTR70 (Sn_C71)	2330	BTR70 (Sn_C71)	1960
Sum	Training Set	6980	Testing Set	13650

**Table 2:** The accuracy of different lengths of RTRBM.

Length of RTRBM	T = 5	T = 10	T = 15	T = 20	T = 25	T = 30
Hidden Units	128	128	128	128	128	128
BMP2	0.5496	0.5556	0.6649	0.6856	0.6900	0.6915
T72	0.7472	0.8345	0.8575	0.8545	0.8723	0.8789
BTR70	0.7594	0.8803	0.9368	0.9402	0.9402	0.9428
Average Accuracy	0.6854	0.7535	0.8197	0.8268	0.8341	0.8377

**Table 3:** Confusion matrix of the model with 384 hidden units.

Methods	Attention Based RTRBM			CRBM (Connected HRRPs)			CRBM (Average HRRP)		
Targets	BMP2	T72	BTR70	BMP2	T72	BTR70	BMP2	T72	BTR70
BMP2	0.9053	0.0717	0.0230	0.8461	0.0821	0.0718	0.8547	0.0819	0.0634
T72	0.0125	0.9758	0.0117	0.0187	0.9726	0.0087	0.0295	0.9516	0.0189
BTR70	0.0347	0	0.9653	0.0448	0.0052	0.9500	0.0525	0.0094	0.9381
Av. Acc.	0.9448			0.9229			0.9157		

## Appendix A

According to Equation (11), we get:

(A1) $$Q_{t+1}=\overset{T}{\underset{\tau =t}{\sum }}h_{\tau +1}W_{hh}{\hat{h}}^{\tau }=Q_{t+2}+h_{t+1}W_{hh}{\hat{h}}^{t}$$

In order to compute $\frac{\partial Q_{t+1}}{\partial {\hat{h}}^{\left(t,m\right)}}$, we need to compute $\frac{\partial Q_{t+1}}{\partial W_{hh}}$ first, which is shown in the equation below.

(A2) $$\frac{\partial Q_{t+1}}{W_{{hh}_{m^{\prime },m}}}=\overset{T}{\underset{t=\tau }{\sum }}\frac{\partial Q_{t+2}}{\partial {\hat{h}}^{\left(t+1,m\right)}}\cdot \frac{\partial {\hat{h}}^{\left(t+1,m\right)}}{W_{{hh}_{m^{\prime },m}}}+\frac{\partial h_{t+1,m^{\prime }}W_{{hh}_{m^{\prime },m}}{\hat{h}}^{\left(t+1,m\right)}}{W_{{hh}_{m^{\prime },m}}}.\;=\overset{T}{\underset{t=\tau }{\sum }}\frac{\partial Q_{t+2}}{\partial {\hat{h}}^{\left(t+1,m\right)}}\cdot {\hat{h}}^{\left(t+1,m^{\prime }\right)}\left(1-{\hat{h}}^{\left(t+1,m^{\prime }\right)}\right){\hat{h}}^{\left(t,m\right)}+\underset{m^{\prime }}{\sum }{\hat{h}}^{\left(t+1,m^{\prime }\right)}{\hat{h}}^{\left(t,m^{\prime }\right)}{}^{T}$$

Therefore, we get:

(A3) $$\frac{\partial Q_{t+1}}{\partial {\hat{h}}^{\left(t,m\right)}}=\overset{T}{\underset{t=\tau }{\sum }}(\frac{\partial Q_{t+2}}{\partial {\hat{h}}^{\left(t+1,m\right)}}\cdot {\hat{h}}^{\left(t+1,m^{\prime }\right)}\left(1-{\hat{h}}^{\left(t+1,m^{\prime }\right)}\right)+h_{t+1,m^{\prime }})\cdot W_{hh}{}_{m^{\prime },m}$$

where

(A4) $$\frac{\partial {\hat{h}}^{\left(t+1,m\right)}}{\partial W_{{hh}_{m^{\prime },m}}}={\hat{h}}^{\left(t+1,m^{\prime }\right)}\left(1-{\hat{h}}^{\left(t+1,m^{\prime }\right)}\right){\hat{h}}^{\left(t,m^{\prime }\right)}{}^{T}$$

is calculated by Equation (4).

According to Equation (A1) and (A4), we get:

(A5) $$\frac{\partial Q_{2}}{\partial W_{{hh}_{m^{\prime },m}}}=\sum_{t=2}^{T}(\frac{\partial Q_{t+1}}{\partial {\hat{h}}^{\left(t+1,m\right)}}\cdot {\hat{h}}^{\left(t,m^{\prime }\right)}\cdot (1-{\hat{h}}^{\left(t,m^{\prime }\right)})+\sum_{m^{\prime }}{\hat{h}}^{\left(t,m^{\prime }\right)})\cdot {\hat{h}}^{\left(t,m^{\prime }\right)}{}^{T}$$

Similarly, the gradients $\frac{\partial Q_{2}}{\partial \Theta }$ can be represented by the equations below.

(A6) $$\left\{ \begin{array}{ll} \frac{\partial Q_{2}}{\partial W_{{hh}_{m^{\prime },m}}} & =\overset{T}{\underset{t=2}{\sum }}(\frac{\partial Q_{t+1}}{\partial {\hat{h}}^{\left(t+1,m\right)}}\cdot {\hat{h}}^{\left(t,m^{\prime }\right)}\cdot (1-{\hat{h}}^{\left(t,m^{\prime }\right)})+\underset{m^{\prime }}{\sum }{\hat{h}}^{\left(t,m^{\prime }\right)})\cdot {\hat{h}}^{\left(t,m^{\prime }\right)}{}^{T}\\ \frac{\partial Q_{2}}{\partial W} & =\overset{T}{\underset{t=1}{\sum }}(\frac{\partial Q_{t+1}}{\partial {\hat{h}}^{\left(t+1,m\right)}}\cdot {\hat{h}}^{\left(t,m^{\prime }\right)}\cdot (1-{\hat{h}}^{\left(t,m^{\prime }\right)})+\underset{m^{\prime }}{\sum }{\hat{h}}^{\left(t,m^{\prime }\right)})\cdot v_{t}^{T}\\ \frac{\partial Q_{2}}{\partial b} & =\overset{T}{\underset{t=1}{\sum }}(\frac{\partial Q_{t+1}}{\partial {\hat{h}}^{\left(t+1,m\right)}}\cdot {\hat{h}}^{\left(t,m^{\prime }\right)}\cdot (1-{\hat{h}}^{\left(t,m^{\prime }\right)})+\underset{m^{\prime }}{\sum }{\hat{h}}^{\left(t,m^{\prime }\right)})\\ \frac{\partial Q_{2}}{\partial b_{0}} & =\frac{\partial Q_{2}}{\partial {\hat{h}}^{\left(2,m\right)}}\cdot {\hat{h}}^{\left(1,m^{\prime }\right)}\cdot (1-{\hat{h}}^{\left(1,m^{\prime }\right)})\\ \frac{\partial Q_{2}}{\partial c} & =0 \end{array}\right.$$

and the gradients $\frac{\partial H}{\partial \Theta }$ are represented below.

(A7) $$\frac{\partial H}{\partial W}=\sum_{t=1}^{T}h_{t}^{T}v_{t};\frac{\partial H}{\partial W_{hh}}=0;\frac{\partial H}{\partial b}=\sum_{t=2}^{T}h_{t};\frac{\partial H}{\partial b_{0}}=h_{1};\frac{\partial H}{\partial W}=\sum_{t=1}^{T}v_{t}$$

## Appendix B

According to Equation (15) and (17), we get:

(A8) $$\frac{\partial F\left(y^{n}|s^{n}\right)}{\partial W_{ys}}=-\frac{1}{\left|D_{train}\right|}\underset{s}{\sum }s_{j}\left(\sigma \left(z\right)-y\right)$$

where $\sigma \left(z\right)=\sigma \left(W_{ys}\cdot s+d\right)$, and $\sigma^{\prime }\left(z\right)=\sigma \left(z\right)\left(1-\sigma \left(z\right)\right)$.

Similarly, we get:

(A9) $$\frac{\partial F\left(y^{n}|s^{n}\right)}{\partial d}=\frac{1}{\left|D_{train}\right|}\underset{c}{\sum }\left(\sigma \left(z\right)-y\right)$$

Submitting Equations (15)–(17) into Equation (14), respectively, the gradients $\frac{\partial F\left(y^{n}|s^{n}\right)}{\partial \theta }$ can be computed exactly, which are shown below.

(A10) $$\frac{\partial F\left(y^{n}|s^{n}\right)}{\partial {\mathrm{Wa}}_{m}}=\frac{\partial F\left(y^{n}|s^{n}\right)}{\partial y\prime_{k}}\cdot \frac{\partial y\prime_{k}^{T}}{\partial s_{i}}\cdot \frac{\partial s_{i}^{T}}{\partial \alpha_{j}}\cdot \frac{\partial \alpha_{j}^{T}}{\partial e_{i}}\cdot \frac{\partial e_{i}^{T}}{\partial {\mathrm{Wa}}_{m}}\;=\frac{\left({y^{\prime }}_{k}-y_{k}\right)}{{y^{\prime }}_{k}\cdot \left(1-y\prime_{k}\right)}\cdot {y^{\prime }}_{k}\cdot \left(1-{y^{\prime }}_{k}\right)\cdot W_{ys}{}_{k,i}^{T}\cdot h_{i,j}^{T}\cdot \frac{\partial \alpha_{j}^{T}}{\partial e_{i}}\cdot {\mathrm{Va}}_{m}\cdot \left[1-tanh^{2}\left({\mathrm{Wa}}_{m}\cdot h_{j}\right)\right]\cdot h_{j}$$

(A11) $$\frac{\partial F\left(y^{n}|s^{n}\right)}{\partial {\mathrm{Va}}_{m}}=\frac{\partial F\left(y^{n}|s^{n}\right)}{\partial y\prime_{k}}\cdot \frac{\partial y\prime_{k}^{T}}{\partial s_{i}}\cdot \frac{\partial s_{i}^{T}}{\partial \alpha_{j}}\cdot \frac{\partial \alpha_{j}^{T}}{\partial e_{i}}\cdot \frac{\partial e_{i}^{T}}{\partial {\mathrm{Va}}_{m}}\;=\frac{\left({y^{\prime }}_{k}-y_{k}\right)}{{y^{\prime }}_{k}\cdot \left(1-y\prime_{k}\right)}\cdot {y^{\prime }}_{k}\cdot \left(1-{y^{\prime }}_{k}\right)\cdot W_{ys}{}_{k,i}^{T}\cdot h_{i,j}^{T}\cdot \frac{\partial \alpha_{j}^{T}}{\partial e_{i}}\cdot tanh\left({\mathrm{Wa}}_{m}\cdot h_{j}\right)$$

where

(A12) $$\frac{\partial \alpha_{j}^{T}}{\partial e_{i}}=\left[\left(1-\beta \right)\frac{-\exp\left(e_{i}+e_{j}\right)}{{\left(\sum_{i}\exp e_{i}\right)}^{2}}+\beta \frac{\exp e_{i}\left(\sum_{i}\exp e_{i}-\exp e_{j}\right)}{{\left(\sum_{i}\exp e_{i}\right)}^{2}}\right]\;\mathrm{with}\;\{\begin{array}{cc} \beta =0, & i\neq j\\ \beta =1, & i=j \end{array}$$
